# Expert consensus on difficulty assessment of endodontic therapy

**DOI:** 10.1038/s41368-024-00285-0

**Published:** 2024-03-01

**Authors:** Dingming Huang, Xiaoyan Wang, Jingping Liang, Junqi Ling, Zhuan Bian, Qing Yu, Benxiang Hou, Xinmei Chen, Jiyao Li, Ling Ye, Lei Cheng, Xin Xu, Tao Hu, Hongkun Wu, Bin Guo, Qin Su, Zhi Chen, Lihong Qiu, Wenxia Chen, Xi Wei, Zhengwei Huang, Jinhua Yu, Zhengmei Lin, Qi Zhang, Deqin Yang, Jin Zhao, Shuang Pan, Jian Yang, Jiayuan Wu, Yihuai Pan, Xiaoli Xie, Shuli Deng, Xiaojing Huang, Lan Zhang, Lin Yue, Xuedong Zhou

**Affiliations:** 1 State Key Laboratory of Oral Diseases & National Center for Stomatology & National Clinical Research Center for Oral Diseases & Department of Operative Dentistry and Endodontics, West China Hospital of Stomatology, Sichuan University, Chengdu, China; 2 Department of Cariology and Endodontology, Peking University School and Hospital of Stomatology & National Center of Stomatology & National Clinical Research Center for Oral Diseases & National Engineering Laboratory for Digital and Material Technology of Stomatology & Beijing Key Laboratory of Digital Stomatology & Research Center of Engineering and Technology for Computerized Dentistry Ministry of Health & NMPA Key Laboratory for Dental Materials, Beijing, China; 3 Department of Endodontics, Shanghai Ninth People’s Hospital, Shanghai Jiao Tong University School of Medicine, College of Stomatology, Shanghai Jiao Tong University, National Clinical Research Center for Oral Diseases, National Center for Stomatology, Shanghai Key Laboratory of Stomatology, Shanghai, China; 4 Department of Operative Dentistry and Endodontics, Hospital of Stomatology, Guanghua, School of Stomatology, Sun Yat-Sen University & Guangdong Provincial Key Laboratory of Stomatology, Guangzhou, China; 5 The State Key Laboratory Breeding Base of Basic Science of Stomatology (Hubei-MOST) & Key Laboratory of Oral Biomedicine Ministry of Education, School and Hospital of Stomatology, Wuhan University, Wuhan, China; 6 Department of Operative Dentistry & Endodontics, School of Stomatology, The Fourth Military Medical University, Xi’an, China; 7 Department of Endodontics, Beijing Stomatological Hospital, School of Stomatology, Capital Medical University, Beijing, China; 8 State Key Laboratory of Oral Diseases & National Center for Stomatology & National Clinical Research Center for Oral Diseases & Department of Preventive Dentistry, West China Hospital of Stomatology, Sichuan University, Chengdu, China; 9 State Key Laboratory of Oral Diseases & National Center for Stomatology & National Clinical Research Center for Oral Diseases & Department of Geriatric dentistry, West China Hospital of Stomatology, Sichuan University, Chengdu, China; 10 Department of Stomatology, First Medical Center, Chinese PLA General Hospital, Beijing, China; 11 Department of Endodontics, School of Stomatology, China Medical University, Shenyang, China; 12 College of Stomatology, Hospital of Stomatology, Guangxi Medical University, Nanning, China; 13 Department of Endodontics, School and Hospital of Stomatology, Nanjing Medical University, Nanjing, China; 14 Department of Endodontics, Stomatological Hospital and Dental School of Tongji University, Shanghai Engineering Research Center of Tooth Restoration and Regeneration, Shanghai, China; 15 Department of Endodontics, Stomatological Hospital of Chongqing Medical University, Chongqing, China; 16 Department of Endodontics, First Affiliated Hospital of Xinjiang Medical University, and College of Stomatology of Xinjiang Medical University, Urumqi, China; 17 Department of Endodontics, Schoolof Stomatology, First Affiliated Hospital of Harbin Medical University, Harbin, China; 18 Department of Endodontics, The Affiliated Stomatological Hospital of Nanchang University, Nanchang, China; 19 Key Laboratory of Oral Disease Research, School of Stomatology, Zunyi Medical University, Zunyi, China; 20 Department of Endodontics, School and Hospital of Stomatology, Wenzhou Medical University, Wenzhou, China; 21 Department of Cariology and Endodontics, Xiangya Stomatological School, Central South University, Changsha, China; 22 Stomatology Hospital, School of Stomatology, Zhejiang University School of Medicine, Zhejiang Provincial Clinical Research Center for Oral Diseases, Key Laboratory of Oral Biomedical Research of Zhejiang Province, Cancer Center of Zhejiang University, Hangzhou, China; 23 School and Hospital of Stomatology, Fujian Medical University, Fuzhou, China

**Keywords:** Pulpitis, Risk factors

## Abstract

Endodontic diseases are a kind of chronic infectious oral disease. Common endodontic treatment concepts are based on the removal of inflamed or necrotic pulp tissue and the replacement by gutta-percha. However, it is very essential for endodontic treatment to debride the root canal system and prevent the root canal system from bacterial reinfection after root canal therapy (RCT). Recent research, encompassing bacterial etiology and advanced imaging techniques, contributes to our understanding of the root canal system’s anatomy intricacies and the technique sensitivity of RCT. Success in RCT hinges on factors like patients, infection severity, root canal anatomy, and treatment techniques. Therefore, improving disease management is a key issue to combat endodontic diseases and cure periapical lesions. The clinical difficulty assessment system of RCT is established based on patient conditions, tooth conditions, root canal configuration, and root canal needing retreatment, and emphasizes pre-treatment risk assessment for optimal outcomes. The findings suggest that the presence of risk factors may correlate with the challenge of achieving the high standard required for RCT. These insights contribute not only to improve education but also aid practitioners in treatment planning and referral decision-making within the field of endodontics.

## Introduction

Endodontic diseases are a group of infectious diseases of the pulpal/periapical tissues caused by a variety of bacteria.^1,2^ Complete eradication of infection from the root canal system is the basis for obtaining a positive outcome.^3,4^ Root canal therapy (RCT) is the recommended alternative.^4,5^ The goal of RCT is to remove infectious agents, cure the disease, and preserve natural teeth. The degree of bacterial infection, the root canal anatomy, the instruments chosen and the treatment techniques employed, are closely related to the success of RCT.^6^ With the intensive research on the bacterial etiology of endodontic diseases, in vitro studies of microfocus CT of the root canal anatomy, and the clinical application of CBCT, clinicians have a better understanding of the anatomy complexity of the root canal system, and the technique sensitivity of RCT, which contributed to the efficacy of treatment.^2,7–13^ RCT efficacy depends on patient compliance, the affected tooth, as well as the technical skills of operators and the equipment of health care institutions. The latter two have nothing to do with the RCT difficulty, but they do with the possibility of solving the difficult cases of RCT. Therefore, this consensus focuses on factors in patients and affected teeth that are directly related to the case difficulty of RCT. How to obtain good outcomes requires pre-treatment assessment of relevant risk factors to avoid complications.

Many studies have focused on the effect of root canal filling on endodontic outcomes.^14–18^ The results of a retrospective study of RCT in China showed that 57.8% of root fillings were of satisfactory quality.^18^ According to the paper based on data from Finland’s Patient Insurance Center, there are 700 dentistry-related claims made annually, with endodontics accounting for 29%.^19^ The explanation could be that RCTs are technically demanding and endodontic cases are beyond the scope of general dentists’ expertise.^20^ Currently, it seems that the more risk factors present, the harder it is to achieve the high standard of root canal therapy required. However, previous studies have not addressed this issue. Therefore, it is essential to analyze and evaluate the difficulty factors of root canal therapy. These insights contribute not only to improve education but also aid practitioners in treatment planning and referral decision-making within the field of endodontics.

## Risk factors for root canal therapy

The first step in treating a patient is to gain a thorough understanding of the patient’s condition and develop an appropriate treatment plan, which first includes a thorough medical examination.^5,21^ Identifying variables that could compromise RCT will help operators prevent possible medical emergencies throughout the procedure.^22^ Following the medical evaluation, an objective examination and x-rays should be completed.^5,22^ Operators can then perform and interpret diagnostic tests to determine a patient’s condition and design high-quality treatment planning that takes into account their needs and preferences. Collecting all the information above avoids misdiagnosis and mistreatment of patients. Proper treatment planning not only helps practitioners avoid procedural pitfalls (e.g., missed root canals, over-removal of dentin, perforation, instrument separation), but also allows dentists to select cases based on their experiences, skills, and comfort levels.^5,23^

### General status of patients

The patient’s own characteristics, including general health status, oral and maxillofacial conditions, and psychosocial status, are closely related to the success of RCT.^5,6,22,24^ These conditions not only determine whether RCT can be performed safely and successfully, but also how difficult it is for operators, which ultimately affect the efficacy of RCT. Chung et al. discovered whatever patient conditions were included or excluded to assess the RCT difficulty, there was a strong positive association between the difficulty and the operating time on all tooth types.^25^ The results of this study imply that in clinical practices, the RCT difficulty in relatively healthy patients depends on the tooth itself. Therefore, it is important to take the patient’s situation into account to fully assess the RCT difficulty. Nevertheless, it is commonly known that RCT gets increasingly difficult for elderly patients for a variety of reasons, including pathology, physiologic aging, and the shrinkage of the pulp canal space brought on by the deposition of secondary and tertiary dentine and cementum^.^^25^

#### Systemic diseases

The patient’s systemic condition influences the choice and implementation of the treatment plan. Systemic diseases not only determine whether RCT can be performed safely, but also affect the RCT outcome.^5,6,22,24^ Before treatment, a comprehensive and detailed medical history should be taken. Multiple criteria for assessing the RCT difficulty use the American Society of Anesthesiologists (ASA) classification to assess the level of risk associated with a patient’s medical history.^23,26^ Emphasis should be placed on asking patients about systemic diseases and medication, including cardiovascular diseases, bleeding disorders, hypertension, diabetes, mental status (with special attention to dental phobia), and history of anesthesia.^27^ Patients’ age, gender, and kind of handicap are other demographic variables that may have an impact on the patient’s overall health.^28^ Nevertheless, several studies have discovered that there is minimal correlation between operating difficulty and the demographic traits of the patients.^25,29^

#### Oral and maxillofacial conditions

Patient-derived factors, including mouth opening restrictions, gagging, salivation, tooth arrangement, and occlusal relationship, are associated with the RCT difficulty and prognosis.^5,20,22,24,25,27,30^ A previous study has reported that more than 40% of the patients experienced limited mouth opening or gaging during RCT.^20^ Among these patient-derived factors, the pharyngeal reflex is closely related to the occurrence of RCT complications.^20^ A previous study has reported that gagging patients experienced noticeably greater complications than non-gagging patients.^20^ Moreover, the ability of mouth opening is closely related to the RCT difficulty.^25,27^ Limited intraoral spaces make it challenging to insert and maneuver intracanal instruments, even with the use of a mouth prop throughout processes.

#### Psychosocial status

Patient demographic factors such as fear, type of caregiver, and oral hygiene maintenance may have a strong relationship with cooperation level, thus affecting the RCT difficulty, especially when patients are dental phobic.^5,20,31^ In addition, some patients may have pain that cannot be resolved using conventional measures, which will make RCT more difficult.^30^

### Tooth conditions

The degree of root canal infection and anatomical diversity of teeth determines the RCT difficulty.^5,6,22,24,32–34^ It is generally accepted that the case difficulty is significantly correlated with the clinical operating time.^25^ Among the many clinical variables that contribute to the RCT difficulty, the tooth anatomical complexity is the main factor that prolongs the operating time in clinical.^25^

#### Infection of dental pulp and root canals

The success of RCT is directly correlated with the state of the dental pulp and the difficulty of debriding the infection in the root canal system.^32–34^ In the early stage of infection, the clearance of the root canal system is easy for viable pulp and non-infected root canals, so the efficacy of RCT is exact.^33,35^ In the late stage of infection, however, especially in the affected teeth with chronic apical periodontitis or post-endodontic diseases, the root canals are severely infected with microbial biofilms.^33,35^ This poses a hard challenge to completely eradicate the infection, particularly in complex anatomical structures including root canal isthmus, lateral branch of root canal, and root canal divergence.^33,36^ Consequently, the effectiveness of RCT is unsatisfactory. Based on the pulp state and the level of root canal infection, root canals are classified into four categories: clean, non-infected, infected, and severely infected root canals.^33^ The elimination difficulty of the root canal system is based on its infection degree.

#### Value of tooth preservation: crown defects and periodontal lesions

Extensive loss of dental hard tissue at the crown leads to reduced fracture resistance and reduced bonding surface, resulting in an inability to hold fillings in place and easy dislodgement, as well as loss of coronal seal.^37^ Periodontal tissue is the supporting tissue for teeth. It is an important part of the chewing function of the tooth. When there is a loss of periodontal tissue due to periodontal disease, it may lead to tooth loss with reduced or even loss of chewing function.^38^ In addition, a number of chronic systemic disorders, most prominently type 2 diabetes, are independently associated with periodontitis.^39^

#### Tooth position in the dentition

The anatomical location of teeth affects the degree of cooperation of the patient, the ease of reaching the affected tooth with instruments, and the difficulty of operators’ maneuvering.^22,27^ Usually, anterior teeth are fully exposed and instruments are easy to enter, thus RCT is less difficult.^27^ Posterior teeth, especially molars, are affected by the patient’s mouth opening and operators’ operating field, so instruments and materials are difficult to enter and RCT is more difficult.^5,20,22,24,27^ Tooth types and positions in the arch were the significant factors affecting operating time and the quality of endodontic treatment outcomes.^25,40^ During micro-endodontics, the maxillary posterior teeth are easier to be observed and operated under the microscope, but the mandibular posterior teeth are more challenging.

#### Tooth eruption position in the dentition

Tooth eruption refers to the process by which teeth gradually emerge from the jawbone to the oral cavity, ultimately attaining a functional occlusal position.^41^ Ectopic eruption may occur as a result of various circumstances.^41,42^ Ectopic eruption encompasses several forms, such as buccal, rotational, and proximal-distal-medial tilted ectopic eruption, which are determined by the position and orientation of teeth and have a significant influence on the degree of RCT difficulty.^22^ For example, the buccal inclination of maxillary molars imposes more difficulty on RCT by limiting the operators’ field of view and increasing the difficulties of the instrument to access cavity preparation. Ectopic eruptions increase the difficulty of rubber dam installation and isolation.

#### Tooth crown morphology and restoration

Abnormalities in tooth morphology result in the variation or loss of important anatomical reference points, thus making it difficult for operators to assess the pulp chamber. Crown morphology is also complicated by development, restoration, or destruction, which influences operators’ judgment of the root canal system.^25,30^ Common clinical conditions include dens invaginatus, prosthesis of the full crown (especially twisted teeth), dental trauma and so on.^23,30^ These conditions increase the incidence of RCT complications. Of the cases where complications occurred, 62% had a wide restoration.^20^ In comparison to patients with normal tooth morphology, patients with abnormal crown morphology also experienced considerably more treatment-related complications.^20^ If access cavity preparation is performed on the prosthesis of the full crown, there is a greater chance of excessive dentin removal and/or perforation because the crown’s orientation may deviate significantly from the root’s orientation.^23^ Moreover, fillings at the tooth cervical region may block the pulp space, which raises the possibility of causing a blockage in the root canal during instrumentation.^23^

### Root canal system configuration

Comprehensive and systematic understanding of the pulp cavity, including the pulp chamber and root canal is important. Pulp chamber, number of canals, shapes and negotiability of canals, and apical closure have a strong relationship with root canal system configuration.^6^ Currently, root canal system configuration is usually obtained clinically by taking preoperative apical radiographs or CBCT.^43–45^

#### Pulp chamber morphology

A receded pulp chamber is caused by tubular secondary or tertiary dentine deposition as a result of pathologic causes (like caries, wedge-shaped defects, restoration) and age-related changes. This deposition manifests itself as matrix deposition along root canal walls, or dentine bridge formation at the orifice of root canals, or complete pulp canal obliteration. Therefore, access cavity preparation and root canal orifice detection are more challenging.^46,47^ The pulp calcification index was proposed to categorize the degree of pulp chamber and root canal calcification.^48^ Grade 1: deep pulp chamber and wide root canal; Grade 2: shallow pulp cavity and thinned root canal with clear root canal imaging; Grade 3: partial disappearance of pulp cavity and root canal; Grade 4: complete disappearance of pulp cavity and root canal. For the purpose of clinical application, pulp chamber calcification was classified into 3 categories. Grade 1: no calcification in pulp chamber; Grade 2: partial calcification in pulp chamber; Grade 3: complete calcification in pulp chamber. In addition, tertiary dentin and dental pulp fillings, such as amalgam core build-up and glass fiber core build-up, can make it more difficult to obtain access to cavity preparation.

#### Number of root canals

In general, the less the number of root canals in a root, the easier the endodontic operation is; on the contrary, when the number of root canals in a root is more, it may result in a smaller diameter of the root canals and more variations in their configuration, thus the more difficult the endodontic operation is.^22,27^ The complexity of root canal topologies inside a single root is determined by the manner in which the main root canal divides along its path from the pulp chamber to the root apex.^49,50^ These configurations include one canal in a single root, two canals in a single root, multiple canals in a single root, three canals in premolars or molars, second mesio-buccal (MB2) canal in maxillary molars, a middle mesial canal in mandibular molars, and atypical root canals.^50–52^ In general, the chance of missing a root canal increases with the number of root canals.^53^ The high percentage of endodontic treatment failure can be ascribed to an untreated missed root canal with bacteria and necrotic tissue inside the canal.^54^ It was revealed that 66.0% of RCT failures in the maxillary first molar involved an untreated MB2 canal.^55^ Moreover, Shah et al. suggested that a tooth with supernumerary roots should be paid extra attention.^56^

#### Root canal morphology

To effectively debride bacteria and necrotic pulp tissue in the root canal, a comprehensive understanding of root canal morphology is necessary.^50^ Based on the root canal morphology, root canals are divided into I-shaped canals, C-shaped canals, J-shaped curve canals, and C/S-shaped curve canals. C/S-shaped curve canal, C-shaped canal system, bifurcating canals in the apical/middle third, and apical delta make root canal negotiability complex, increase the risk of creating a blockage or separating an instrument in canals, and complicate obturation of the canal space.^23,57–60^ A C-shaped canal system was likely to present in the fused root tooth, and the prevalence is 39% in Chinese mandible second molars. C-shaped canals appeared to divide into two or more canals towards the canal terminus.^61^ Isthmuses within the root canal system, may contain necrotic debris, tissue remnants, or organic substrates that support the growth of microorganisms, leading to the difficulty of orthograde root canal instrumentation, debridement and root filling of canal isthmuses.^62,63^ A small canal that branches off of the main root canal and typically connects to the external surface of a root or furcation is known as an accessory canal. As such, it can occur anywhere in the pulp chamber (chamber canals) or throughout the entire root canal wall (coronal, middle, or apical third).^64^ For the Chinese population, the prevalence of accessory canals is 52.5%, and among them, 86.5% were found in the apical third, 12.8% in the middle third, and 0.7% in the coronal third of the root canal.^65^ Anywhere along the root, complicated structural features that interact with peri-radicular tissues facilitate the spread of bacteria and their byproducts, which can cause periodontitis.^66,67^

#### Root canal curvatures

Root canal curvature is one of the key indicators used to assess the RCT difficulty.^68^ Formation of step, root canal deviation, and instrument separation are the most common complications during the negotiation and shaping of a curved root canal.^69^ In clinical practice, radiological examinations are usually taken to determine the root canal curvature and evaluate the RCT difficulty before surgery. Based on the curvature of root canals measured by Schneider’s method, the calculated curvature of root canal is divided into three categories: straight root canals (0–10 degrees of root canal curvature), moderately curved root canals (10–30 degrees of root canal curvature), and severely curved root canals (more than 30 degrees of root canal curvature).^22,70,71^ In addition, the radius and length of the root canal curvature also affect the RCT difficulty.^22,27,72^ At the same bending angle, the smaller the curved radius, the more difficult it is. The longer the distance between the apical stop and the root canal bend, the more difficult it is to bend the instruments at the curved point and, consequently, the greater the chance of instrument separation.^27^

#### Root canal length

The root canal length determines the endodontic working length, which is one of the most crucial variables in endodontic preparation.^8^ The working length of a root canal is of utmost importance in keeping the preparation inside the restricted radicular space and determines the operating length of gutta-percha, in order to avoid apical extrusion and secure good obturation.^8^ Too long or too short root canal length increases RCT difficulty.^27^ A previous study revealed that the longer the root canal length, the more difficult it is to fill the canal, and the lower the likelihood of obtaining tight obturation.^73^ The root canal length is typically 16–25 mm; any length <16 mm is referred to as the very short teeth, while any length longer than 25 mm is referred to as the very long teeth.^73^

#### Root canal calcification

Root canal calcification is determined primarily on the basis of radiological examination, in conjunction with root canal preparation.^27^ Both age-related changes and endodontic/periodontal-related diseases cause physiologic or pathologic calcification in the root canal system. Root canal calcification is characterized by the deposition of calcified tissue along the canal walls.^74^ As a result, the pulp chamber and root canal space can become partially or completely obliterated, resulting in a receded pulp chamber, narrow root canal, and even apical blockage, which increases the risk of procedural errors during root canal preparation. These errors include transportation, ledges, perforations, instrument separation, and alterations of the internal anatomy.^75,76^ Based on the degree of calcification, root canal calcification is divided into three categories.^22,27^ Class I: visible canals, no obvious calcification in root canals, and access to the physiological apical foramen smoothly by the first file; Class II: obscuring canals, scattered calcification in root canals, and access to the physiological apical foramen by the first file after canal negotiation; Class III: blurring canals, obvious calcification in root canals, and difficult detection of the root canal orifice. In addition, the diameter of a root canal, the initial endodontic K-file size, and the position of root canal calcification all pose difficulty to root canal therapy. Therefore, combining the imaging manifestations and the initial file sizes, we classify root canal calcification into three categories. Grade I: canals clearly visible in radiographs or easy access to physiological foramen with 15# K file; Grade II: pulp chamber/canals visible with volume reduced or irregular shape or pulp stone located in the center in radiographs or access to physiological foramen with 10# K file; Grade III: pulp chamber/canals almost indistinctive or canals invisible and unclear or pulp stone located above canal orifice in radiographs or access to physiological foramen with 8# K file.

#### Root resorption (including internal, external and apical root resorption)

Tooth root resorption is linked to both physiological and pathological conditions, leading to the progressive destruction of cement, dentin, or bone tissues and, ultimately, tooth loss.^77,78^ Based on clinical and radiographic manifestations, root resorption can be diagnosed. Nonetheless, a complete examination of the patient is necessary since patients suffering from root resorption frequently have minimal or no clinical symptoms.^56^

#### Development of root

The development stage of the root is closely related to the diameter of the apical foramen.^79^ In young permanent teeth, root canals are oversized, especially in the apical 1/3. The apical foramen is flared without apical stop. During RCT, instruments, infected substances and root canal filling materials tend to beyond the apical foramen, damaging periapical tissues and causing infections or re-infections in periapical area.^22,27^ In the case of a root that has been apically amputated due to an apical cyst, the apical stop may be destroyed, making RCT more difficult.^46^

### Root canal retreatment

Retreatment access has been referred to as coronal disassembly because the previous coronal and radicular restorations are necessary to be disassembled or removed.^80^ Most teeth have a full-coverage restoration after the initial RCT, frequently with a post and core in place. Coronal-radicular access for retreatment is significantly more difficult in these cases when compared to endodontically treated teeth that have been minimally restored. Root canal retreatment is often accompanied by endodontics mishaps such as canal blockages, ledges, and destruction of apical stops.^78^ These mishaps prevent instruments from reaching the working length or facilitate files beyond the apical constriction, causing apical underfilling or overfilling.^81,82^ More complications occurred in the patients who had experienced complications in previous RCTs.^20^ In addition, the root canal system in the endodontic post-treatment cases is usually infected severely. A precious study has reported that if the root canal morphology was previously changed, the overall success rate was reported to be 47% at a 2-year follow-up.^73^

#### Crown restoration

Crown restorations including direct and indirect restorations restore the shape of the tooth crown and the occlusal relationship. Direct restorations, including resin fillings and amalgam fillings, pose difficulty to access cavity preparation and orifice locating. In general, because of the large color difference between amalgam fillings and dental hard tissues, amalgam fillings are easy to be distinguished, which has little effect on the access cavity preparation and orifice locating. However, the color of resin fillings and dental hard tissues are proximate. It is very difficult to distinguish them, which poses difficulty to root canal therapy. Full crown, inlay, onlay, and overlay are all common indirect restorations. Among them, a post-and-core crown is one of the most common restorations for teeth after RCT.^83^ Tooth structure has always been altered in endodontic retreatment, and is commonly quite misrepresentative of the original anatomy of the tooth. In most cases where old restorations are simply removed, retreatment difficulty has little correlation to restorations. Unfortunately, retreatment may be more difficult when restorations are in situ since restricted visibility may raise the risk of an iatrogenic mishap.^84^ Furthermore, it will be more difficult to remove canal obstructions like posts, and there is a greater possibility that the clinician may overlook something crucial like a fracture, an additional canal, or hidden recurrent caries.^84^ Therefore, depending on the restoration materials and whether or not restorations were removed, we categorized the RCT difficulty into three categories. Grade I: amalgam fillings and the removal of indirect restorations; Grade II: resin fillings; Grade III: indirect restorations.

#### Posts

Posts are commonly utilized in the restoration of endodontically treated teeth, hence a post is very common to be encountered when preparing the access during retreatment.^85,86^ There are a wide variety of posts the clinician may encounter during retreatment. The shape, design, and material of posts and the length of posts in root canals are closely associated with retention force in root canals, which have some influence on the operators’ ability to remove them. In addition, adhesive materials used to cement posts, tooth types and locations in the arch also influence post-removal.^86,87^ For the location, the more posterior the tooth in the arch, the more difficult the post is to be removed. This predicament is a result of accessibility. The more accessible the tooth is, the easier the post is to be removed since the clinician will have more techniques and instruments available to use.^88^ Additionally, the opposing occlusion will not impede post-removal as much as the further anterior the tooth is. Depending on post materials and the length of posts in root canals, we categorized the RCT difficulty into three categories. Grade I: cast post and its length in root canals <1/2; Grade II: cast post and its length in root canals more than 1/2 or fiber post and its length in root canals less than 1/2; Grade III: fiber post and its length in root canals more than 1/2.

#### Materials and quality of root canal filling

Removing the previous root-filling materials is the prerequisite to regain access to the apical area in endodontic retreatment. This part is complicated by the large variety of types of root-filling materials used, such as silver points, phenol-soaked paper points, bioceramic materials, and gutta-percha. It has been reported that it is very difficult to regain access to the apical area when the root-filling materials are silver points or carrier-based obturations.^30^ It is crucial to ascertain the type of root filling to minimize surprises when attempting retreatment. Compared to the others, gutta-percha is relatively easy to be removed with a combination of heat, solvents, and mechanical instrumentation.^80,89^ The length and quality of the filling also influence the ease of gutta-percha removal.^20^ It is minimally difficult to regain access to the apical area when there is no root canal obstruction.^6^ It is easy to regain access to the apical area in the previously treated teeth with short, poorly condensed root fillings and evidence of probable canal patency beyond existing root filling.^30^ However, it is greatly difficult to regain access to the apical area in the teeth with well-condensed root fillings to length or overfilled roots (more than 2 mm) with apical lesions.^17,21,30,90^

#### Instrument separation

An instrument may occasionally separate during RCT, resulting in a poorly cleaned root canal system that may compromise the outcome of treatment.^91^ This instrument is usually some type of file. The presence of a separated instrument in the canal system may be detected during retreatment immediately upon diagnosis, or it may become apparent until the root-filling materials are removed. The incidence of hand instrument separation has been reported to be 0.25% and for rotary instruments, it ranges from 1.68% to 2.4%.^92,93^ Currently, stainless steel K files and nickel-titanium rotary files are more frequently used in clinical. Compared with stainless steel K files, nickel-titanium files are more flexible, but it is very easy for secondary instrument separation to occur when removing separated nickel-titanium files, thus greatly increasing the RCT difficulty. For this reason, we uniformly classified the cases in which a nickel-titanium instrument separation occurred in root canals as Grade III. Although a variety of tools are available for the removal of instruments separated in the root canals, not all of them can be successfully removed. Several complications may occur during the process, such as excessive tooth structure removal, perforations, and so on.

#### Anatomic ledges

It is a type of canal transportation that results in a canal irregularity on the outside of the canal curvature that is difficult or impossible to bypass.^94,95^ Posttreatment disease is often associated with ledges because the canal space apical to the ledge is not adequately cleaned and sealed.^94^ Retreatment cases often present with previous endodontic mishaps such as blockages and ledges in the apical portion of canals. Most of these ledges are iatrogenic mishaps resulting from vigorous instrumentation short of the appropriate working length and failure to confirm apical patency regularly during instrumentation.

#### Perforations

Occasionally, the posttreatment endodontic disease will be the result of root perforation.^96^ Root perforations are created pathologically by resorption and caries, and iatrogenically during RCT. Frequently, cervical and occasionally mid-root perforations are associated with epithelial down growth and subsequent periodontal defects, thus making a tight seal difficult to achieve.^91,97^

## Criteria for assessing RCT difficulty

So far, several assessment criteria have been proposed to assess RCT difficulty. These assessment criteria are based on the characteristics of their country’s population and are somewhat geographically specific.

Among them, the Endodontic Case Difficulty Assessment Form (ECDAF) published by the American Association of Endodontists (AAE) is the first assessment criteria for assessing RCT difficulty. The criteria include six patient considerations, eight diagnostic and treatment considerations, and three additional considerations, and categorize the RCT difficulty into three levels: minimal, moderate, and high. Based on the criteria, the AAE developed specific criteria in 2006 to evaluate the risk factors and make them quantifiable.^23^ The criteria make case selection more efficient, more consistent, and easier to document, as well as containing more comprehensive patient information. It basically reflects the RCT difficulty, thus being widely recognized and highly recommended.

The Canadian Academy of Endodontics (CAE) also proposed a system for evaluating the RCT difficulty. It included a total of 16 risk factors, including four patient considerations, nine tooth considerations, and three additional considerations.^98^ Each risk factor was categorized into three degrees and specifically quantified to assess the RCT difficulty. Compared with the AAE criteria, for the first time, this assessment system included the resin post in the pulp chamber and iatrogenic factors. However, it is not suitable for generalization in clinical practice due to the relatively large number of assessment indicators.

The Dutch endodontic treatment index (ETC) consists of two parts: the endodontic treatment index scale and the endodontic treatment difficulty scale. The endodontic treatment index includes 15 risk factors. Compared with the AAE and CAE, ETC enables clinicians to rapidly assess cases that are easy to perform. If some additional conditions are found intraoperatively, the RCT difficulty should be reassessed. For some cases with high operational difficulty, a second assessment is required, which increases the clinician’s workload.^26^

One common feature of AAE, CAE, and Dutch ETC is that the complexity indices were integrated and assigned a cumulative numerical value, which increases with the degree of complexity.^6^

Taking into account the actual situation in China, the anatomical characteristics of Chinese root canals, the in-depth study of the etiology of endodontic and periapical diseases, the complexity of root canal anatomy, and the rapid development of clinical technology, we propose a Chinese endodontic case difficulty assessment criteria (ECDA) to assess the RCT difficulty. The criteria not only include the endodontic treatment difficulty assessment form (Table 1) but also the difficulty level classification of root canal therapy (Table 2). The Chinese ECDA contains four parts: patient condition, tooth condition, root canal system configuration, and root canal needing retreatment, which classifies the RCT difficulty into four levels. Each risk factor is categorized into three grades: 1, 2 and 3.

**Table 1:** Endodontic therapy difficulty assessment form

Criteria	Grade 1 difficulty	Grade 2 difficulty	Grade 3 difficulty
A *Patient considerations*			
1. Medical history/anesthesia	□No medical problem (ASA Class 1*)	□Basic-controlled medical problem (ASA Class2*)	□Complex medical history/consultation of physicians (ASA Class3*) □pregnant or lactating women □Hard to achieve anesthesia
2. Maximum mouth opening	□Three grown-up’s finger width in opening	□Two grown-up’s finger width in opening	□One grown-up’s finger width in opening
3. Gag reflex	□None	□Occasional	□Serious
4. Dental phobia	□Cooperative	□Anxious but cooperative	□Uncooperative
5. Oral hygiene maintenance	□Good	□Acceptable	□Poor
6. Diagnosis	□Mild pain or swelling □Typical signs and symptoms: clear diagnosis □Minimal difficulty in obtaining/interpreting radiographs	□Moderate pain or swelling □Differential diagnosis of usual signs and symptoms □Moderate difficulty in obtaining/interpreting radiographs (e.g., high floor of mouth, narrow or low palatal vault)	□Severe pain or swelling □Complex signs and symptoms: difficult diagnosis □Extreme difficulty in obtaining/interpreting radiographs (e.g., overlapped anatomical structures)
B *Tooth conditions*			
7. Periodontal lesions	□None or mild periodontal disease	□Concurrent moderate periodontal disease	□Mobility/deep periodontal pocket/perforation/gingival cleft □Furcation involvement □Combined endodontic/periodontic lesion □Root resection/hemisection required
8. Infection degree	Limitation in pulp chamber (irreversible pulpitis)	Limitation in main root canal and no biofilms (irreversible pulpitis, necrotic pulp)	Spread to whole root canal system or external root surface, biofilms (chronic apical periodontitis)
9. Tooth position in the dentition	□Anterior/premolar	□1st molar	□2nd or 3rd molar
10. Tooth eruption position in the dentition	□Moving into eruption space exactly □Slight inclination (<10°) □Slight rotation (<10°)	□Moderate inclination (10°–30°) □Moderate rotation (10°–20°)	□Extreme inclination (>30°) □Extreme rotation (>20°)
11. Tooth crown morphology and restoration	□Normal original crown morphology □Crown axis consistent with root axis	□Macrodontism /microdontism □Crown/root moderate variation □Extensive crown defect	□Fused tooth /dens invaginatus □Difference between crown and root axis □Full coverage restoration/filling
12. Root conditions	□ One root	□Two roots	□Three roots in mandible molars □Three more roots □Developmental grooves of root surface (including palato-gingival grooves, apical development grooves) □Fused roots □Root bifurcation (coronal, middle, apical)
C *Root canal configuration*			
13. Pulp chamber morphology	□No calcification in pulp chamber □Normal access	□Partial calcification in pulp chamber □Amalgam core build-up in pulp chamber □No canal post	□Complete calcification in pulp chamber □Porcelain fused to metal, metal, porcelain crown □Glass fiber core build-up in pulp chamber □Canal post/cast post and core
14. Number of root canals	□One canal in a single root □Anterior tooth or premolar with 1 canal	□Two canals in a single root □Anterior tooth or premolar with 2 canals □Molar with ≤3 canals	□Multiple canals in a single root □Premolar with 3 canals □Molar with å 3 canals □Second mesio-buccal (MB_2_) canal in maxillary molar □Middle mesial canal in mandibular molar □Atypical root canals
15. Root canal morphology	□I-shaped canal	□J-shaped curve canal □Previously started, but not completed endodontic treatment □Oval shaped canal	□C/S-shaped curve canal □C-shaped canal system □Canal branch in middle or apical third □Isthmus, bifid canals in middle or apical third □ A canal with a ledge, abrupt curve canal,
16. Root canal curvatures	□0°–10°	□10°–30°	□>30°
17. Root canal length	□16–25 mm	□<16 or 25–30 mm	□≥30 mm
18. Root canal calcification	□Canals clearly visible in radiographs □Easy access to physiological foramen with 15 # K file	□Pulp chamber/canals visible, with volume reduced, irregular shape in radiographs □Pulp stone located in the central □Access to physiological foramen with 10 # K file	□Pulp chamber/canals almost indistinctive □Canals invisible and unclear in radiographs □Pulp stone located above the canal orifice □Access to physiological foramen with 8 # K file difficultly
19. Root resorption	□No resorption	□Slightly apical resorption, apical morphology unbroken	□External resorption □Internal resorption □Extensive apical resorption
20. Development of root	□Apex closed (≤0.3 mm in diameter)	□Apex opening (0.3–1.2 mm in diameter), irregular shape	□Apex opening(≥1.2 mm in diameter), irregular shape
D *Root canal needing retreatment*			
21. Crown restoration	□Routine dismantling of plastic restorations, crown and bridges □ Amalgam fillings and the removal of indirect restorations	□Wide or full coverage restorations □ Resin fillings	□Indirect restorations
22. Posts	□No canal post □Cast post and its length in root canals less than 1/2	□less than 8 mm □Cast post and its length in root canals more than 1/2 or fiber post and its length in root canals less than 1/2;	□More than 8 mm □Posts are thought to be associated with a perforation □Fiber post and its length in root canals more than 1/2.
23. Materials and quality of root canal filling	□No root canal obstruction □Short, poorly condensed root fillings □Evidence of probable canal patency beyond existing root filling	□Short, well condensed root canal fillings □warm vertical condensation □single cone root canal filling	□ Well condensed root canal fillings to length or overfilled (more than 2 mm) □Root-filling materials are silver points or carrier-based obturations
24. Instrument separation	□No instrument separation □Separation location at coronal third root canal □Separation length less than 2 mm	□Separation location at middle third root canal □Separation length 2–3 mm	□Separation location at apical third root canal □Separation length >3 mm □Ni–Ti rotary file
25. Perforations	□No perforation	□Perforation location at chamber floor or coronal third canal	□Strip perforations □Perforation location at the apical third canal
26. CBCT assessment	□No canal isthmus □Far distance between mandibular posterior apex and inferior alveolar nerve canal □Far distance between maxillary posterior apex and maxillary sinus floor	□No canal isthmus □3 mm distance between mandibular posterior apex and inferior alveolar nerve canal □3 mm distance between maxillary posterior apex and maxillary sinus floor	□Canal isthmus □Touched or approached distance between mandibular posterior apex and inferior alveolar nerve canal □Touched or approached distance between maxillary posterior apex and maxillary sinus floor
Whether make referral decision		□Yes	□No
Referral reasons:			
Dentist:		Time:

**Table 2:** Difficulty level classification of endodontic therapy

	Difficulty level classification
Level I	All risk factors are assessed into grade 1.
Level II	Only one risk factor is assessed into grade 2, and the remaining are assessed into grade 1.
Level III	Two or more risk factors are assessed into grade 2, or one is assessed into grade 3.
Level IV	Two or more risk factors are assessed into grade 3.

If all risk factors are assessed into grade 1, the RCT difficulty is referred to as Level I. If just only one risk factor is assessed into grade 2 and the remaining are assessed into grade 1, the RCT difficulty is referred to as Level II. If two or more risk factors are assessed into grade 2, or one is assessed into grade 3, the RCT difficulty is referred to as Level III. If two or more risk factors are assessed into grade 3, the RCT difficulty is referred to as Level IV.

Endodontic cases referred to as Level I can be performed competently by undergraduates and postgraduate students who have just begun endodontic specialist training. If the RCT difficulty is referred to as Level II, endodontic cases should be operated by a physician with extensive clinical experience or an endodontic postgraduate student who is in the second year of a master’s degree under the supervision of a specialist. If the RCT difficulty is referred to as Level III or IV, endodontic cases should be referred to an endodontist or a postgraduate endodontic specialist in a higher grade.

## Description of the application of the assessing criteria

### Education and training

Many endodontic mishaps occur in relation to the operator’s own lack of knowledge, skills and experience.^99^ The RCT quality is also related to the operator’s training experience.^100,101^ It is essential for undergraduate students to receive theoretical and skill training.^46^ The analysis of endodontic therapy difficulty factors can be used to train undergraduate students to form a knowledge framework of pulpal/periapical diseases and acquire preclinical skills, to help clinicians systematically assess various complexity factors and determine RCT difficulty levels, as well as to recommend whether to make patient referrals to achieve triage in primary and secondary care.^56^

#### Education for undergraduate students

The analysis of Endodontic therapy difficulty factors can be used to develop student’s knowledge structure for information gathering and analysis prior to pulpal/periapical treatment. All students participating in an undergraduate clinic should be required to evaluate case difficulty preoperatively.^91^ According to the endodontic undergraduate curriculum requirements, the clinical evaluation exercise holds significant value in assessing students’ knowledge and abilities.^25,102^

#### Preclinical training

When students and supervisors assess the difficulty categorization of the RCT by the endodontic case difficulty assessment form (ECAF) presented in the Finnish current care guidelines for endodontic treatment (2014), 46% of the complications appeared in cases that student judged the level of RCT difficulty to be lower than teachers, compared with 14% of the cases that students assessed the identical RCT difficulty with teachers.^20^

When assessing RCT difficulty levels for the same tooth with the same assessing criteria, there are differences between students and teachers. In the pilot study, the assessments by students and the supervisor differed in 55% of cases, especially in moderately difficult cases. In the majority of these cases (71%), the students evaluated the case to be easier than the teacher.^20^ The AAE reported that using an endodontic case assessment form improves dental students’ ability to evaluate the RCT difficulty more effectively than not using one.^5^ Preclinical training, therefore, instructs students to correctly and effectively assess RCT difficulty levels and ensures that everything the student does is within his or her ability and comfort zone.

#### Clinical training

Studies have shown that root canal therapy has a high success rate.^35,103^ However, due to the significant differences in knowledge, skills and experience between general dentists and endodontic specialists, some operators may be not sufficient to handle some challenging cases, which can lead to a high risk of procedural errors.^56^ Usually, the operator’s own stress level is also closely related to the complexity of the case.^29^ Only when the operator’s skills and expertise match the technical requirements, treatment should be started.^23,104^ It is reasonable to select suitable cases of root canal treatment for different operators.^6^

Treatments in dentistry, like other surgical fields, are dependent on available resources. Graded diagnosis and treatment are critical for arranging clinical resources including staff, operating space, and facilities.^25^ Even for specialized practitioners, it is anticipated that complex cases will take more time and effort to complete.^25^ Several studies have shown that the percentage of satisfactory root canal fillings performed by undergraduate dental students, postgraduate students, and general dental practitioners is less than 50%. However, the research conducted by endodontists to evaluate the quality of root canal fillings revealed that more than 77% of the fillings exhibited a high level of technical quality.^105^ It is clear that specialized endodontics are preferable for handling more challenging endodontic cases.^40^ When an endodontic case has several complicating variables or one factor that makes RCT extremely difficult, a referral to an endodontic expert is recommended. Although the decision to refer a patient to an endodontic specialist depends on the skills and experience of the referring dentist, a preoperative assessment of RCT difficulty can assist operators in making a referral decision.^20^ Digital products such as EndoApp and its adapted vision—the BES EndoApp have been widely used in Europe and the United States to clinically assess RCT difficulty and to assist dentists to take therapeutic measures on patients or make referrals.^46,56^

### Clinical applications

#### Preoperative RCT difficulty analysis and development of treatment plans

It should choose the suitable technique and method for root canal therapy based on the evaluation of the RCT difficulty.

#### Doctor-patient-communication

More precise and accurate patient information regarding the treatment plan, potential complications, limitations, and associated expenses can be provided to patients after the RCT difficulty has been assessed.

#### Instructions for solving difficult problems and preventing complications

Performing a formal case assessment before treatment initiation may preempt challenges by recognizing possible problems. This lowers the likelihood of iatrogenic damage and treatment failure and also upholds the important principle of ‘first do no harm’.

#### Prediction of curative effect

Information from the analysis of Endodontic case difficulty factors may help guide discussions with patients regarding the challenges in achieving a predictable outcome, the ideal environment to complete treatment, and the financial expenses and risks to be considered whether or not a referral is required.^106,107^

## Conclusions and expectations

A comprehensive endodontic case difficulty assessment criteria is an important guide for clinicians to formulate a treatment plan, evaluate the efficacy of treatment, communicate effectively with patients, and minimize medical disputes during the initial consultation. After comprehensively examining the overall role of the vast majority of objective anatomical factors and non-anatomical risk factors, these assessment criteria are effective in distinguishing different RCT difficulties. In general, the more difficult the root canal treatment, the lower the success rate. To improve the RCT success rate, it is advisable that if the RCT difficulty is degree III or IV, endodontic cases should be referred to an endodontist or a postgraduate endodontic specialist in a higher grade. This assessment criteria can not only be used to assess the RCT difficulty, but can also provide the referral basis for highly difficult cases, and it is profitable to establish a comprehensive referral system for endodontic treatment of endodontic and periapical diseases.
